# Factors Impacting Video Telehealth Appointment Completion During COVID-19 Pandemic Among People Living with HIV in a Community-Based Health System

**DOI:** 10.1007/s10461-021-03394-7

**Published:** 2021-07-26

**Authors:** Nicole Ennis, Laura Armas, Seyram Butame, Hemali Joshi

**Affiliations:** 1 Department of Behavioral Sciences and Social Medicine, Florida State University, 2010 Levy Ave, Bldg. B, Suite 266, Tallahassee, FL 32310 USA; 2 CAN Community Health, Sarasota, FL USA

**Keywords:** COVID-19 pandemic, HIV, Telehealth, Digital disparities

## Abstract

As the threat of COVID-19 on vulnerable populations continues, mitigation protocols have escalated the use of telehealth platforms, secure 2-way video platforms with audio capabilities. The goal of the current study was to examine factors associated with successful completion of video telehealth appointments in HIV care. We utilized a random effects logistic model to assess characteristics of patient encounters that predicted completed telehealth visits. Results show that factors such as identifying as black (AOR = 0.30, 95% CI 0.23–0.40, p < 0.01), identifying as heterosexual (AOR = 0.40, 95% CI, 0.29–0.55, p < 0.01), identifying as Hispanic/Latinx (AOR = 0.67, 95% CI, 0.48–0.95), having public insurance (e.g., Ryan White funding, Medicare/Medicaid) (AOR = .25, 95% CI 0.19–0.33, p < .001), and having detectable viral load (AOR = .049, 95% CI, 0.31–0.76) are negatively associated with completion of telehealth appointments. Results suggest that greater efforts to address the digital divide are needed to increase access to video telehealth.

**Supplementary Information:**

The online version contains supplementary material available at 10.1007/s10461-021-03394-7.

## Resumen

A medida que la amenaza de la pandemia de Covid-19 continúa en poblaciones vulnerables, los protocolos para mitigarla han intensificado el uso de plataformas seguras de audio y video bidireccional, conocidas como plataformas de telesalud. El objetivo del estudio actual es el examinar los factores asociados con el logro de completar las consultas por video de telesalud en la atención de personas que viven con VIH. Utilizamos un modelo logístico de efectos aleatorios para evaluar las características de los encuentros con los pacientes, para predecir si una consulta de telesalud se completó. Los resultados muestran que los factores asociados con una consulta de telesalud completa incluyen identificarse de raza negra (RMA = 0.30, 95% IC 0.23–0.40, p < 0.01), etnicidad de hispano/ latinx (RMA = 0.67, 95% IC 0.48–0.95), tener seguro de salud público, por ejemplo fondos de Ryan White, Medicare o Medicaid (RMA = 0.25, 95% IC 0.19–0.33, p < 0.01) y tener una carga viral detectable (RMA = 0.49, 95% IC 0.31–0.76, p < 0.01). Estos resultados sugieren que se necesitan mayores al para abordar el problema de la brecha digital para aumentar el acceso a la plataforma de telesalud.

## Introduction

Access to medical treatment and care for those living with or at risk for HIV is vital to ensuring quality of life and limiting the spread of the disease [1–3]. For people living with HIV (PLWH), lack of access to medical care is associated with poor disease management, antiretroviral medication failure, and increased incidence of ER visits and/or hospitalizations [4–6]. Further those on pre-exposure prophylactic (PrEP) therapy need regular monitoring of liver enzymes and renal functioning to ensure mitigation of long-term toxic effects [7–9]. Regular monitoring of those on PrEP also ensures early detection of other sexually transmitted infections and allows for monitoring of HIV status [10]. As the threat of COVID-19 on vulnerable populations continues, mitigation protocols have escalated the use of telehealth platforms, secure 2-way video platforms with audio capabilities, for the safe management of uninterrupted care [11–13]. CAN Community Healthcare (CAN), a community-based nonprofit organization providing prevention services and care to PLWH and those at high risk, has been at the forefront of adaptive telehealth services for this population.

To address the medical care needs of patients living with HIV providers have created telehealth protocols that allow them to examine, assess, and treat patients. Telehealth has been successfully implemented in many healthcare systems throughout the US for a wide range of patient populations [13–15]. Since the onset of the COVID-19 pandemic, many providers have described telehealth as a successful means for providing medical care [16, 17]. However, little is known about factors impacting access to telehealth in general and specifically for those living with or at risk for HIV. Telehealth platforms require that both the patient and the provider have high speed internet connections and are using adequately powered devices to maintain connection for the entire appointment. While telehealth has been successfully implemented for a wide range of patients in routine care, factors impacting access to telehealth care has not been closely examined.

The goal of the current study was to examine factors associated with successful completion of video telehealth appointments. To that end, we examined patients with or at risk for HIV who were scheduled for video telehealth appointments at CAN community health clinics during the first 6 months of active telehealth care implementation. We hypothesized that demographic factors such as race, ethnicity, and insurance status (a proxy for socioeconomic status) would be adversely associated with successful completion of video telehealth appointments.

## Methods

CAN Community Health, is a private, not-for-profit organization dedicated to serving the needs of people living with or at risk for HIV using a comprehensive, multidisciplinary approach to healthcare. Over 90% percent of CAN Community Health clinics are located throughout Florida. In March of 2020 CAN Community Health transitioned to telehealth appointments for most patients. The CAN EMR was already enabled for Telehealth visits due to a pre-planned (i.e. before the pandemic) systemwide rollout, scheduled for later in the year. Due to the urgency of the pandemic, rollout occurred in mid-March 2020 and the institutional COVID-19 task force developed a phased plan to implement mitigation strategies and limit in-person visits, based on availability of PPE, local infection rates, staff need for medical or dependent care accommodations, and government-level mandates, such as lock-downs. Clinics were geographically aligned into regions to provide access for critical needs and staff rotation. A tiered medical necessity protocol for in-person visits to address special needs for PLWH, HCV, and STIs protocol was adapted from the Centers for Medicare & Medicaid Services (CMS) guidance and implemented by the clinic medical director and practice administrator, and centralized oversight. Patients were seen for routine medical care, assessment for PrEP, and HIV testing. We used data from patients who received a telehealth visits between April 1, 2020 and October 31, 2020 at 24 sites within the CAN Community Health System. April 1, 2020 was chosen as the start date to ensure that all clinics had implemented the telehealth platform and troubleshooted technical challenges. Data for this study was taken from the CAN Community Health electronic medical record (EMR), eClinicalWorks®. Data of arrived and no-show visits with providers ranging from April 1, 2020 to October 31, 2020 were the parameters used to extract the initial dataset. Resources like labs, nurses, peers, etc. were then excluded. There were no filters applied for clinical service type and/or provider specialty. Visit type and provider specialty were included with an HIV flag column and each patient’s last viral load and CD4 values were included. A PrEP flag column was also included with a count of arrived visits (not filtered for clinical service type and/or provider specialty). All data was de-identified prior to data analysis.

## Measures

### Primary Outcome

*Telehealth Medical Appointment Completion*: was defined as visits completed via 2-way video-audio connection. Completed visits were coded as 1 = completed. Incomplete telehealth visits (i.e., patients were unable to connect via 2-way video and audio and reverted to phone only) were coded as 0 = incomplete.

### Predictors

*Demographics*: Race, ethnicity, age, sexual orientation, and gender are self-reported variables extracted from the EMR.

*HIV viral Load*: is defined as undetectable ≤ 200 copies/mL (coded as 0) or detectable > 200 copies/mL (coded as 1) based on most recent HIV-1 RNA test value in the EMR.

*CD4 Count*: is defined as normal > 200 copies/mm (coded as 0) or below normal < 200 copies/mm (coded as 1).

*Insurance Status*: has four groups; private insurance, public insurance, other, and no information. Private insurance denotes health plans not run by federal or state governments (coded as 1). Public insurance denotes those health plans that are state or a federal government operated (coded as 2). The other category captures alternative means by which patients pay for services, such as out-of-pocket and hospital or clinic write-offs through charity (coded as 3). The last group denotes encounters with no information (coded as 4).

*Adherence*: is a categorical variable with two groups, strict adherence, and moderate adherence. The first group characterizes patients with no missed visits during the 7-month period from which data was extracted. The latter group characterized patients with one or more missed visit occurring during that 7-month period. Appointment adherence as operationalized as the ratio of appointments missed out of the total number scheduled, following the methods of Catz et al. (1999) [18].

## Data Analysis

Statistical analyses were performed using Stata v16.0. Prior to analyses, assumptions of normality and linearity were evaluated and screening was conducted for outliers. No outliers or significant skewness were detected; therefore, variables were left untransformed. Univariate frequency distributions and means with standard deviations were used to describe categorical and continuous variables, respectively. Bivariate correlations were calculated to determine the associations among predictor variables and telehealth medical appointment adherence. The encounter data reported was treated as an unbalanced panel data set. Furthermore, a random effects logistic model was utilized to assess the specific characteristics of patient encounters that predicted completed video telehealth visits. Effect sizes with a p-value of less than 0.05 were assessed as statistically significant.

## Results

### Description of Patients

From April 1, 2020, through October 31, 2020, 4873 individuals sought and completed treatment services from various CAN Community Health locations. During that same period, there were 11,006 separate care and treatment encounters across all sites. The number of encounters ranged from a single visit to 34 separate encounters over the study period. The mean number of visits was 2.32 (SD = 2.35). At their last encounter 2642 patients completed a video telehealth visit, 1046 completed a phone-only visit, and the remainder, 1185, completed an in-person encounter. See Table 1 for information on demographic characteristics of the patient population at their last encounter with a CAN site. About a third of the patients had private insurance (30%) and 94% of the patients were established patients at their last complete encounter. From the perspective of race and ethnicity, most patients identified as white (~ 55%) and while a minority identified as being of Hispanic origin (~ 16%). Lastly, in terms of HIV, most had HIV under control with about 94% having a viral load that was undetectable and most having a CD4 cell count that was normal (i.e., more than 200 cell/mm^3^ of blood).

**Table 1:** Characteristics of individuals attending care and treatment visits at CAN Community Health (N = 4873; 11,006 encounters)

Variable	n (%)/min–max, m (sd)
Age	16–88 years 48.69 (13.64)
Sex	
Female	1032 (21.2)
Male	3839 (78.8)
Gender	
Female	965 (20.2)
Male	3675 (70.1)
Transgender/genderqueer	93 (2.0)
Other	34 (0.7)
Orientation	
Lesbian/gay/homosexual	2609 (56.6)
Heterosexual	1598 (34.7)
Bisexual	221 (4.8)
Other	181 (3.9)
Race	
Black/African American	1502 (31.2)
White	2632 (54.6)
Other	683 (14.2)
Ethnicity	
Not Hispanic/Latino	3712 (83.8)
Hispanic/Latino	716 (16.2)
HIV status	
Negative	593 (12.2)
Positive	4,280 (87.8)
CD4-count	
Normal (> 200 cells/mm3)	3753 (95.6)
Below Normal (< 200 cells/mm3)	172 (4.4)
Viral load	
Undetectable (< 200 copies/mL)	3723 (93.8)
Detectable (> 200 copies/mL)	245 (6.2)
Established patient	
No	290 (6.0)
Yes	4583 (94.0)
Insurance status	
Private insurance	1118 (30.3)
Public insurance	2352 (63.7)
Other	166 (4.5)
No Information	56 (1.5)
Adherence	
Strict adherence	4376 (89.8)
Moderate adherence	497 (10.2)

### Bivariate Analysis

Adherence behavior was calculated for all 4873 unique CAN patients from the 7-month sample. Of the sampled patients with at least one scheduled and completed visit during the study period, most (89.9%) were strictly adherent to their appointments meaning they had no missed visits (See Table 2). Adherence behavior exhibited a non-normal distribution and was thus categorized into two groups, strict adherence (no missed visits) and moderate adherence (at least one missed visit). Patients' use of video telehealth was not independent of adherence behavior (χ^2^ = 14.1, p < 0.01).

**Table 2:** Factors associated with video telehealth visits in CAN Community health patients (N = 4873; 11,006 encounters)

		Non-video visit (n = 2231)	Video telehealth (n = 2642)	Χ^2^/t-test, p
Age		50.6 (SD = 13.8)	47.1 (SD = 13.3)	9.2; < 0.01
Sex	Female	573	459	50.3; 0.01
	Male	1656	2183	
Gender	Female	544	421	58.3; 0.1
	Male	1572	2103	
	Transgender/genderqueer	46	47	
	Other	13	21	
Orientation	Lesbian/gay/homosexual	933	1676	213.3; < 0.01
	Heterosexual	935	663	
	Bisexual	100	121	
	Other	97	84	
Race	Black/African American	899	603	176.5; < 0.01
	White	1016	1616	
	Other	294	389	
Ethnicity	Not Hispanic/Latino	1740	1972	6.3; 0.01
	Hispanic/Latino	299	417	
HIV status	Negative	201	392	38.4; < 0.01
	Positive	2030	2250	
CD4-count	Normal (> 200 cells/mm3)	1671	2082	14.5; < 0.01
	Below Normal (< 200 cells/mm3)	102	70	
Viral load	Undetectable (< 200 copies/mL)	1633	2090	36.4; < 0.01
	Detectable (> 200 copies/mL)	156	89	
Established patient	No	129	161	0.2; 0.6
	Yes	2102	2481	
Insurance status	Private insurance	358	760	230; < 0.01
	Public insurance	1338	1014	
	Other	45	121	
	No information	40	16	
Adherence	Strict adherence	1964	2412	14.1; < 0.01
	Moderate adherence	267	230	

### Multivariate Analysis

As noted above, multivariate analysis (See Table 3) was limited to patients scheduled for and completing at least one health care visits during the study period. Patients identifying as black had lower odds (AOR = 0.30, 95% CI 0.23–0.40, p < 0.01) of successfully completing a video telehealth visit compared to patients identifying as white. Patients who identified as Hispanic/Latinx also had lower odds (AOR = 0.67, 95% CI, 0.48–0.95, p = 0.02) of completing a successful video telehealth appointment. Similarly, patients on public insurance (e.g., Ryan White funding, Medicare/Medicaid) had lower odds (AOR = 0.25, 95% CI 0.19–0.33, p < 0.01) of completing video telehealth visits, compared to patients on private health insurance plans. Those with a detectable viral load (AOR = 0.049, 95% CI, 0.31–0.76), those who identify as heterosexual (AOR = 0.40, 95% CI, 0.29–0.55, p < 0.001), and older patients (AOR = 0.96, 95% CI, 0.95–0.97, p =  < 0.01) all had lower odds of completing video telehealth visits over the study period.

**Table 3:** Predictors of completed video telehealth visits (A random effects model of N = 4873; 11,006 encounters)

	AOR	SE	p-value	95% CI	
Age	0.96	0.00	< 0.01**	0.95	0.97
Gender identity					
Female (referent)					
Male	0.56	0.36	0.36	0.16	1.94
Transgender/genderqueer	0.73	0.53	0.67	0.17	3.05
Other	0.29	0.28	0.20	0.05	1.87
Sex (at birth)					
Female (referent)					
Male	1.21	0.75	0.76	0.36	4.09
Sexual Orientation					
Lesbian, gay or homosexual (referent)					
Heterosexual	0.40	0.07	< 0.01**	0.29	0.55
Bisexual	0.71	0.21	0.23	0.40	1.25
Other	0.39	0.14	0.01*	0.19	0.77
Race					
White (referent)					
Black/African American	0.30	0.04	< 0.01**	0.23	0.40
Other	2.10	0.51	< 0.01**	1.31	3.38
Ethnicity					
Not Hispanic (referent)					
Hispanic/Latino	0.67	0.12	0.02*	0.48	0.95
HIV Status					
Negative (referent)					
Positive	0.75	0.38	0.57	0.28	2.02
Viral Load					
Undetectable (referent)					
Detectable	0.49	0.11	< 0.010**	0.31	0.76
Insurance status					
Private insurance (referent)					
Public insurance	0.25	0.04	< 0.01**	0.19	0.33
Other	0.56	0.21	0.13	0.26	1.18
No information	0.08	0.03	< 0.01**	0.04	0.16
Adherence					
Moderate adherence (referent)					
Strict adherence	1.31	0.20	0.08	0.97	1.77

*p-value ≤ 0.05; **p-value ≤  0.01

## Discussion

This study sought to examine the factors associated with scheduling and completing video telehealth appointments. Results suggest that video telehealth appointments are a feasible alternative to in-person health visits for a certain subset of patients living with or at risk for HIV and is associated with optimal appointment adherence. However, there is clear evidence that not all patients in the CAN Community Health system have access to optimal telehealth services (i.e., 2-way secure audio and video digital connection) that allow healthcare professionals to visually examine, assess, and treat patients in the course of a routine visit. In the current sample, approximately one-third of all video telehealth appointments were discontinued due to the patient’s lack of digital access needed to complete the appointment. Factors associated with unsuccessful completion of a video telehealth appointment include: race, ethnicity, insurance status, viral load, and sexual orientation. These results support our hypothesis that race, ethnicity, and insurance status are primary factors associated with unsuccessful video telehealth appointment completion in this patient population. In the current study insurance status was used as a marker of socioeconomic status. Previous studies have demonstrated that patients of lower socioeconomic status and racial and ethnic minorities are more likely to miss HIV care appointment and have worse clinical outcomes [2, 4, 6, 19, 20]. In the current study, patients’ inability to connect to video telehealth appointments due to technology barriers led to incomplete video telehealth appointments that became phone-only appointments. Phone only appointments limit healthcare professionals’ ability to visually assess patient complaints. Patients were adherent to their medical appointments and attempted to connect to the appointments but were then forced to convert the video telehealth appointment to phone-only appointments due to technology difficulties. This demonstrates one way the digital divide can limit the range of services available to those without the technology needed to access 2-way secure video and audio platforms. Those who often have the most to gain from telehealth approaches are also the least likely to have access to broadband [21, 22] and/or cannot afford the necessary technology. Currently, approximately three-in-ten adults with household incomes below $30,000 a year do not own a smartphone, and more than four-in-ten do not have home broadband services (44%) or a computer (46%) [22] Additionally, at least 162 million Americans and 1.4 million Native Americans living on Tribal lands [23] have little-to-no broadband access. The digital divide can be characterized as lack of access to a high-speed internet connection and the personal computers and smart phones needed to use the connection [24]. Lack of access to digital resources is generally due to a combination of factors such as: lack of access to broadband due to geographical location, lack of access to technology needed such as lower-performance computer, lower speed wireless connections, and lower-priced connections such as dial up [25, 26].The lack of access is problematic because of the rise in services such as video telehealth that require access to high speed reliable networks using adequately powered devices.

Additional factors associated with completion of video telehealth appointments that we did not account for include age, viral load, and sexual orientation. Previous literature has found that older age is associated with greater challenges related to the use of digital services [27]. While older adults are not a monolithic group as it relates to internet usage, within a range of demographic sub-categories such as race, education, income, and sex, older adults have lower rates of internet usage compared to younger adults [28]. In the current sample, the finding that HIV status and sexual orientation are were associated with not completing video telehealth visits is likely due to socioeconomic resources. Patients who are HIV negative are seen for PrEP services which requires self-pay or private insurance in most cases, indicating greater socioeconomic resources. The finding that heterosexuals were less likely to complete a video telehealth appointment is unclear and likely due to a combination of factors such as race and socioeconomic status, and that heterosexual men tend to be less engaged in care [1]. While in this sample, those identifying as lesbian, gay, or homosexual were more likely to be white and have private insurance indicative of greater socioeconomic resources.

The finding that detectable viral load is associated with not completing a video telehealth appointment is supported by prior literature [13, 29, 30]. In the US those of lower socioeconomic status are more likely to have detectable viral load due to lack of access to needed care [19, 31]. While video telehealth appointment completion is not sufficient to achieve optimal treatment outcomes, it is a component of HIV care that may help improve clinical outcomes. Therefore, the relationship between unsuccessful video telehealth appointment completion and viral load suggests that access to care is an essential component to achieving optimal disease management. Results suggest that efforts are needed to increase access to video telehealth care and are warranted because it improves access to care.

Results of the current study should be viewed in light of certain study limitations. First, these analyses were based on EMR data, which is observational, and therefore cannot be used to establish causality. Second, the data reflects encounters of which the majority (92%) occurred in the state of Florida, which had a different approach than other states regarding lockdown restrictions. Generally, Florida eased lockdown restrictions faster than other states; therefore, patients returned to in clinic appointments at a faster rate (see supplementary Fig. 1 for details). Due to lack of power we were unable to run state by state comparisons. Finally, because patient income data was not available in the EMR data, insurance status was used as a proxy for socioeconomic status. Despite the limitations this study has noteworthy strengths. Study strengths include the use of a large community clinic encounter dataset with a diverse population. Additionally, due to the swift response of the CAN Community Health team, we were able to obtain data from the start of the pandemic and telehealth services in April 2020 through October 2020 when restrictions eased in Florida and throughout much of the country. Further, this study examined a wide range of income and racial ethnic backgrounds due to the CAN Community Health population served.

## Conclusion

The COVID-19 pandemic has fundamentally changed the ways care can be delivered to patients; however, this requires that we are mindful of socioeconomic differences in access to care. Digital telehealth pods in centralized locations have the potential to quickly address digital disparities for telehealth services, in particular for those who do not have access to broadband in their homes and/or lack the needed device and data plan. Further, partnerships with large national level retail pharmacies that provide healthcare services are another opportunity to expand services via digital kiosks. Currently, CAN Community Health is developing and implementing the CAN Connect program which places computers in collaborating agencies to assist with provider visits, case management, patient care coordination services. Currently in the US, there is no research on the effectiveness of digital telehealth pods on increasing access to healthcare services. Increasing access to video telehealth platforms may be a more easily modifiable target for improving the benefits of routine HIV care. HIV treatment requires a lifetime commitment and making care more accessible for all is one way to improve health outcomes. Interventions to increase digital access to underserved populations are needed in order to ensure that video telehealth is a reliable platform to deliver HIV care to all patients.

## Supplementary Information

Below is the link to the electronic supplementary material.

Supplementary file1 (DOCX 16 kb)
