# Supplementary material for: Factors Impacting Video Telehealth Appointment Completion During COVID-19 Pandemic Among People Living with HIV in a Community-Based Health System
Source: AIDS Behav. 2021 Jul 26;26(2):407–14. doi: 10.1007/s10461-021-03394-7 (PMC8313002; doi:10.1007/s10461-021-03394-7)
Supplement: Supplementary file 1 — Supplementary file1 (DOCX 16 kb) [file 10461_2021_3394_MOESM1_ESM.docx]

**Figure 1** Total number of encounters by study month and by encounter type from April 1, 2020 to October 31, 2020 CAN Community Health.
